# Tocolytic action and underlying mechanism of galetin 3,6-dimethyl ether on rat uterus

**DOI:** 10.1186/s12906-017-2007-6

**Published:** 2017-12-02

**Authors:** Juliana da Nóbrega Carreiro, Iara Leão Luna de Souza, Joedna Cavalcante Pereira, Luiz Henrique César Vasconcelos, Rafael de Almeida Travassos, Barbara Viviana de Oliveira Santos, Bagnólia Araújo da Silva

**Affiliations:** 1 Programa de Pós-graduação em Produtos Naturais e Sintéticos Bioativos (PPgPNSB), Universidade Federal da Paraíba (UFPB), João Pessoa, Paraíba Brazil; 2 Centro de Biotecnologia, Universidade Federal da Paraíba (UFPB), João Pessoa, Paraíba Brazil; 3 Departamento de Ciências Farmacêuticas, Universidade Federal da Paraíba (UFPB), João Pessoa, Paraíba Brazil; 4 Centro de Ciências da Saúde/Pós-Graduação em Produtos Naturais e Sintéticos Bioativos/Laboratório de Farmacologia Funcional Prof. George Thomas, Universidade Federal da Paraíba, Cidade Universitária, P.O. Box 5009, João Pessoa, Paraíba 58051-970 Brazil

**Keywords:** Galetin 3,6-dimethyl ether, Flavonoid, Tocolytic action, Ion channels, Rat uterus

## Abstract

**Background:**

Galetin 3,6-dimethyl ether (FGAL) is a flavonoid isolated from aerial parts of *Piptadenia stipulacea*. Previously, FGAL was shown to inhibit both carbachol- and oxytocin-induced phasic contractions in the rat uterus, which was more potent with oxytocin. Thus, in this study, we aimed to investigate the tocolytic action mechanism of FGAL on the rat uterus.

**Methods:**

Segments of rat uterus ileum were suspended in organ bath containing modified Locke-Ringer solution at 32 °C, bubbled with carbogen mixture under a resting tension of 1 g. Isotonic contractions were registered using kymographs and isometric contractions using force transducer.

**Results:**

FGAL was more potent in relaxing uterus pre-contracted with oxytocin than with KCl. Additionally, FGAL shifted oxytocin-induced cumulative contractions curves to the right in a non-parallel manner, with E_max_ reduction, indicating a pseudo-irreversible noncompetitive antagonism of oxytocin receptors (OTR) or a downstream pathway target. Moreover, FGAL shifted CaCl_2_-induced cumulative contraction curves to the right in a non-parallel manner in depolarizing medium, nominally without Ca^2+^, with E_max_ reduction, suggesting the inhibition of Ca^2+^ influx through Ca_V_. The relaxant potency of FGAL was reduced by CsCl, a non-selective K^+^ channel blocker, suggesting positive modulation of these channels. Furthermore, in presence of apamin, 4-aminopyridine, glibenclamide or 1 mM TEA^+^, the relaxant potency of FGAL was attenuated, indicating the participation of SK_Ca_, K_V_, K_ATP_ and highlighting BK_Ca_. Aminophylline, a non-selective phosphodiesterase (PDE) blocker, did not affect the FGAL relaxant potency, excluding the modulation of cyclic nucleotide PDEs pathway by FGAL.

**Conclusion:**

Tocolytic effect of FGAL on rat uterus occurs by pseudo-irreversible noncompetitive antagonism of OTR and activation of K^+^ channels, primarily BK_Ca_, leading to calcium influx reduction through Ca_V_.

## Background

Natural products have played an important role in the process of discovery and drug development [1]. These products and their derivatives represent more than 50% of all drugs in clinical use worldwide, and plants contribute no less than 25% of this total [2]. In this context, several natural products have been isolated from species of the Fabaceae family, including benzofuranoids, essential oils, triterpenoids, alkaloids and flavonoids [3].

This family includes a plant found in the northeastern Brazilian province of Caatinga, the species *Piptadenia stipulacea* (Benth.) Ducke, popularly known as “Jurema-branca” [4], “Jurema-malícia-da-serra”, “Caracará” and “Calumbi” [5]. This species is widely used in folk medicine for treatment of inflammatory processes, being consumed as a decoction or tincture of its barks and leaves [6].

**Fig. 1:**
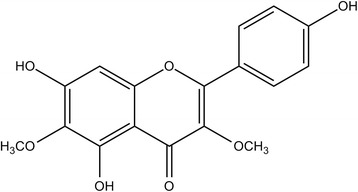
Chemical structure of flavonoid galetin 3,6-dimethyl ether (FGAL) [8]

Flavonoids are low molecular weight phenolic compounds, secondary metabolites found in plants [7]. A flavonoid named galetin 3,6-dimethyl ether (FGAL) (Fig. 1) was isolated from the chloroform phase of the crude ethanolic extract obtained from the aerial parts of *Piptadenia stipulacea* [8]. This flavonoid presented interesting biological activities, such as antiviral [9], anticancer [10] and antioxidant [11]. In addition, the flavonoid presented spasmolytic activity on guinea pig ileum and trachea and rat aorta and uterus [12].

In recent years and in light of the vital importance of myometrium in physiological processes, there has been remarkable progress in the understanding of uterine pathophysiology, such as embryo implantation and disorders such as dysmenorrhea and colic, which occurs by an uncontrolled uterine contractions process [13]. The exaggerated contractions culminate in pain, even leading to reduced uterine vascular flow, hypoxia and ischemia, further increasing the level of pain [14].

Some flavonoids have presented spasmolytic activity in rat uterus, such as genistein, kaempferol, quercetin [15], and isoliquiritigenin, which also showed analgesic activity in mice [16], and FGAL [12], which also presented anti-inflammatory and anti-nociceptive activities in mice [8].

Therefore, we decided to investigate the tocolytic action mechanism of FGAL on rat uterus given the importance of flavonoids as secondary metabolites produced by plants, in addition to the fact that substances able to exert tocolytic action are promising for treating uterine pain associated with smooth muscle contraction dysregulation, such as uterine colic and dysmenorrhea.

## Methods

### Animals

Virgin female Wistar rats (40 rats, 150–250 g) were used for all experiments. The animals were maintained under standard conditions with a 12 h light/dark cycle in a temperature-controlled environment (21 ± 1 °C). They had free access to water and food (Purina®, Brazil). All experimental procedures were performed in accordance with the guidelines approved by the Animal Research Ethics Committee (CEPA) of Laboratório de Tecnologia Farmacêutica (LTF)/Universidade Federal da Paraíba (UFPB) (protocol n° 0303/11).

### Chemicals

FGAL was obtained as previously described [8]. Briefly, FGAL was isolated from the aerial parts of *Piptadenia stipulacea* Benth., species collected in the city of Serra Branca, Paraíba, Brazil. The sample were identified by PhD. Maria de Fátima Agra, from UFPB. A voucher, Agra et al. 3331 (JPB) was deposited in the Herbarium Lauro Pires Xavier in the Departamento de Sistemática e Ecologia from UFPB (João Pessoa, PB, Brazil).

Potassium chloride (KCl) and calcium chloride bi-hydrate (CaCl_2_.2H_2_O) were purchased from Merck & Co. Inc. (Whitehouse Station, NJ, USA). Apamin, cesium chloride (CsCl), tetraethylammonium chloride (TEA-Cl), glibenclamide, 4-aminopyridine (4-AP) and diethylstilbestrol were purchased from Sigma-Aldrich Co. (Saint Louis, MO, USA). Oxytocin was purchased from Eurofarma (Brasil). All substances were dissolved in distilled water, except glibenclamide and diethylstilbestrol, which were dissolved in ethanol PA (95%). FGAL was solubilized in Cremophor EL® (3%) plus distilled water. The final concentration of Cremophor EL® in the organ bath never exceeded 0.01% (*v*/v), which does not produce any observable effect on rat uterine tonus.

### Tissue preparation and measurement of contractile tension

Rats were pretreated with diethylstilbestrol 1.0 mg/kg (s.c.) 24 h prior to the estrus induction experiment. The animals were sacrificed by cervical dislocation. The rat uterus was immediately removed, cleaned of connective tissue and fat and immersed in Locke-Ringer solution (in mM: NaCl, 154.0; KCl, 5.63; CaCl_2_.2H_2_O, 2.16; MgCl_2_.6H_2_O, 2.10; glucose, 5.55; and NaHCO_3_, 5.95) [14] and continuously bubbled with a carbogenic mixture (95% O_2_ and 5% CO_2_). A depolarizing Locke-Ringer solution (60 mM KCl), nominally without Ca^2+^, was also used with KCl in equimolar exchange for NaCl [15]. The segment of uterus was cut longitudinally into strips (1 to 2 cm in length and approximately 1 mm wide). Then, the strips were suspended by cotton thread in organ baths at 32 °C. The isotonic contractions were recorded on a drum of a smoky kymograph using lever, and the isometric contractions were registered by a force transducer TIM-50 (São Paulo) coupled to a data acquisition system (AECAD 04F, AQCAD 2.0.3., AVS Projects, SP). The uterus segments were stabilized with a 1.0 g resting tension (baseline) by at least 40 min. At this time, the solution was changed every 10 min.

### Experimental procedures

#### FGAL effect on KCl- or oxytocin-induced tonic contractions

After the stabilization period, two similar concentration-response curves for 60 mM KCl or 10^−2^ IU/mL oxytocin (OXY) were obtained, and after the plateau phase of the second contraction, cumulative concentrations of FGAL were added to the organ bath to obtain a relaxation curve. The results were expressed as the reverse percentage of initial contraction elicited by contractile agents. The pEC_50_ value of FGAL was calculated and compared to both contractile agents.

#### FGAL effect on oxytocin-induced cumulative contractions

After the stabilization period, two similar cumulative concentration-response curves for OXY (10^−5^-3 × 10^−1^ IU/mL) were obtained (control), and then, in the presence of different concentrations of FGAL pre-incubated for 15 min, a third cumulative concentration-response curve for OXY was obtained. The maximal contraction obtained with the control concentration-response curve to OXY was taken as 100%, and all concentration-response curves in the presence of FGAL were calculated as a function of this value. pEC_50_ value of OXY was calculated in both absence and presence of FGAL concentrations. The antagonism exerted by FGAL was analyzed based on the values of Schild slope [17] and its potency on the pEC_50_ value.

#### FGAL effect on CaCl_2_-induced contractions in depolarizing medium (60 mM KCl) nominally without Ca^2+^

After the preparations were stabilized, Locke-Ringer solution was replaced by a depolarizing Locke-Ringer solution, nominally without Ca^2+^. After 30 min, 60 mM KCI was added to produce smooth muscle depolarization and remained in the bath throughout the experiment. Ten min after KCI addition, two similar cumulative concentration-response curves for CaCl_2_ (3 × 10^−6^–10^−2^ M) were obtained (control), and a third cumulative concentration-response curve for CaCl_2_ was then obtained in the presence of different concentrations of FGAL pre-incubated for 15 min. The E_max_ obtained with the control concentration-response curve for CaCl_2_ was taken as 100% and served as the reference for all concentration-response curves assessed in the presence of FGAL [15].

#### FGAL effect on oxytocin-induced tonic contractions in the absence and presence of K^+^ channel blockers

In uterine segments, some pharmacological tools were incubated for 20 min before the contraction induced by the contractile agent to investigate the participation of different types of K^+^ channels, as follows: 5 mM CsCl, non-selective K^+^ channel blocker [18]; 3 mM 4-AP, voltage-gated K^+^ channel (K_v_) blocker [19]; 3 × 10^−5^ M glibenclamide, ATP-sensitive K^+^ channel (K_ATP_) blocker [20]; 100 nM apamine, small-conductance Ca^2+^-activated K^+^ channel (SK_Ca_) blocker [21]; or 1 mM TEA^+^, big-conductance Ca^2+^-activated K^+^ channel (BK_Ca_) blocker [22]. After stabilization of the tonic contraction induced by 10^−2^ IU/mL OXY, cumulative concentrations of FGAL were added to the bath to obtain the relaxation curve. The results were expressed as the reverse percentage of initial contraction elicited by OXY. The relaxation potency of FGAL was evaluated by comparing the pEC_50_ values in both the absence and the presence of blockers.

#### FGAL effect on oxytocin-induced tonic contractions in the absence and presence of non-selective phosphodiesterase inhibitor

Aminophylline (10^−4^ M), a non-selective phosphodiesterase (PDE) inhibitor [23], was added to organ baths for 20 min followed by an OXY-induced tonic contraction, and FGAL was then cumulatively added to obtain the relaxation curve. The results were expressed as the reverse percentage of initial contraction elicited by OXY, and the relaxation potency of FGAL was evaluated by comparing the pEC_50_ values in both the absence and the presence of aminophylline.

### Statistical analysis

All results were expressed as mean ± standard error of mean (SEM). Student’s t-test, for single comparisons and one-way ANOVA followed by Bonferroni’s post-test for multiple comparisons were used in the data analysis, and results were considered significant when *P* < 0.05. Curves and pEC_50_ values were calculated by non-linear regression and Schild slope and pEC’_50_ (negative logarithm to base 10 of molar concentration value of an antagonist that reduces the response to an agonist to 50% of its maximum effect) by linear regression [17]. All analyses were performed using GraphPad Prism® software version 5.01 (GraphPad Software Inc., San Diego, CA, USA).

## Results

### FGAL effect on KCl- or oxytocin-induced tonic contractions

**Fig. 2:**
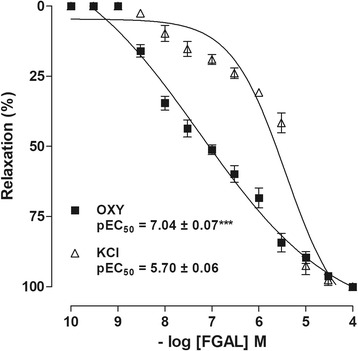
FGAL effect on tonic contractions induced by KCl or oxytocin in rat uterus. FGAL effect on tonic contractions induced by 60 mM KCl (□) or 10^−2^ IU/mL oxytocin (■) in rat uterus (*n* = 5). Symbols and vertical bars represent the mean and SEM. Student’s t test, ***P < 0.001 (KCl vs. oxytocin)

In a concentration-dependent manner, FGAL (10^−9^–10^−4^ M, *n* = 5) relaxed the uterus pre-contracted with 60 mM KCl (pEC_50_ = 5.7 ± 0.06) or 10^−2^ IU/mL oxytocin (pEC_50_ = 7.0 ± 0.08) (Fig. 2). An analysis of the pEC_50_ values indicates that FGAL was more potent in inhibiting the contractions induced by oxytocin. After the control experiments, all preparations showed complete reversion of the contractile response within 2 h.

### FGAL effect on oxytocin-induced cumulative contractions

**Fig. 3:**
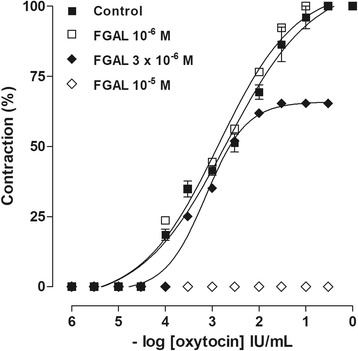
Cumulative-contractions response curves to oxytocin in absence and presence of FGAL in rat uterus. Cumulative-contractions response curves to oxytocin in the absence (■) and presence of FGAL 10^−6^ (△), 3 × 10^−6^ (▲) and 10^−5^ M (◇) in rat uterus (*n* = 5). Symbols and vertical bars represent the mean and SEM

FGAL 10^−6^ M did not present effect, however FGAL (3 × 10^−6^ and 10^−5^ M, *n* = 5) inhibited the cumulative concentration-response curves to oxytocin. These curves were shifted to the right in a non-parallel manner, with decreasing E_max_ (Table 1, Fig. 3). The FGAL pEC_50_’ was 5.04 ± 0.02, and Schild slope value was 0.75 ± 0.16.

**Table 1:** E_max_ and EC_50_ values of oxytocin-cumulative contractions curves in absence and presence of FGAL (n = 5)

[FGAL] M	E_max_ (%)	EC_50_ (M)
Control	100.0	2.7 ± 0.1
10^−6^	100.0	2.9 ± 0.02
3 × 10^−6^	65.4 ± 0.7^***, ###^	3.1 ± 0.02^**^
10^−5^	0.2 ± 0.04^***, ¥¥¥^	Nd

Data are expressed as the mean ± S.E.M. (n = 5)

One-way ANOVA followed by Bonferroni’s post-test

^**^ *P* < 0.05 and ^***^ *P* < 0.001 (FGAL vs. control); ^###^ *P* < 0.001 (10–6 vs. 3 × 10–6 FGAL); ^¥¥¥^ *P* < 0.001 (3 × 10–6 vs. 10–5 FGAL). *ND* not determined

### FGAL effect on CaCl_2_-induced contractions in depolarizing medium, nominally without Ca^2+^

**Fig. 4:**
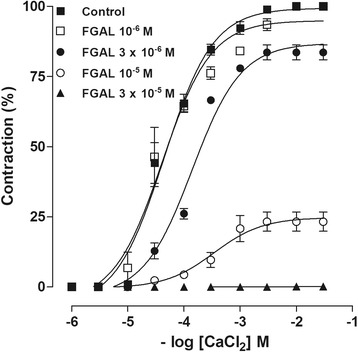
Cumulative-contractions response curves to CaCl_2_ in absence and presence of FGAL in rat uterus. Cumulative-contractions response curves to CaCl_2_ in depolarizing medium, nominally without Ca^2+^, in the absence (■) and presence of FGAL 10^−6^ (□), 3 × 10^−6^ (●), 10^−5^ (○) and 3 × 10^−5^ M (▲) in rat uterus (*n* = 5). Symbols and vertical bars represent the mean and SEM

In depolarizing Locke-Ringer solution, nominally without Ca^2+^, (60 mM KCl), FGAL (3 × 10^−6^, 10^−5^ and 3 × 10^−5^ M, *n* = 5) significantly reduced the CaCl_2_-induced maximal contractile response and promoted a concentration-dependent rightward shift of CaCl_2_ concentration-response curves (Table 2, Fig. 4).

**Table 2:** E_max_ and EC_50_ values of CaCl_2_-cumulative contractions in absence and presence of FGAL (n = 5)

[FGAL] M	E_max_ (%)	EC_50_ (M)
Control	5.5 ± 0.4 × 10^−5^	100.0
10^−6^	6.2 ± 0.6 × 10^−5^	100.0
3 × 10^−6^	1.5 ± 0.05 × 10^–4***, ###^	83.6 ± 1.2^***, ###^
10^−5^	3.5 ± 0.4 × 10^–4***, ¥¥¥^	23.3 ± 1.5^***, ¥¥¥^
3 × 10^−5^	ND	0.17 ± 0.03^***, §§§^

Data are expressed as the mean ± S.E.M. (n = 5)

One-way ANOVA followed by Bonferroni’s post-test

^***^ *P* < 0.001 (FGAL vs. control); ^###^ *P* < 0.001 (10–6 vs. 3 × 10–6 FGAL); ^¥¥¥^ *P* < 0.001 (3 × 10–6 vs. 10–5 FGAL); ^§§§^ *P* < 0.001 (10–5 vs. 3 × 10–5 FGAL). *ND* not determined

### FGAL effect on oxytocin-induced tonic contractions in the absence and presence of K^+^ channels blockers

**Fig. 5:**
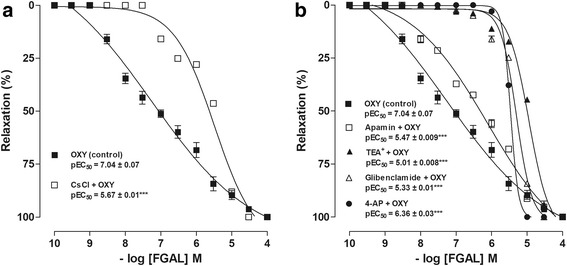
FGAL effect on tonic contractions induced by oxytocin in absence and presence of K^+^-channels blockers. FGAL effect on tonic contractions induced by 10^−2^ IU/mL oxytocin in both absence (■) and presence of CsCl (□) (**a**), 4-AP (▼), apamin (◇), 1 mM TEA^+^ (▼) or glibenclamide (○) (**b**) in rat uterus (n = 5). Symbols and vertical bars represent the mean and SEM, respectively. Student’s t test, ***P < 0.001 (OXY vs. blockers + OXY)

FGAL (10^−9^–10^−4^ M, *n* = 5) completely relaxed the uterus pre-contracted with 10^−2^ IU/mL oxytocin (pEC_50_ = 7.0 ± 0.08), but its relaxant potency was attenuated after pre-incubation with 5 mM CsCl (pEC_50_ = 5.7 ± 0.01) (Fig. 5a). The relaxant potency of FGAL was attenuated in the presence of specific K^+^ channel blockers (Fig. 5b): 3 mM 4-AP (pEC_50_ = 5.5 ± 0.01), 30 μM glibenclamide (pEC_50_ = 5.3 ± 0.01), 1 mM TEA^+^ (pEC_50_ = 5.0 ± 0.009) and 100 nM apamine (pEC_50_ = 6.4 ± 0.03).

### FGAL effect on oxytocin-induced tonic contractions in the absence and presence of aminophylline

**Fig. 6:**
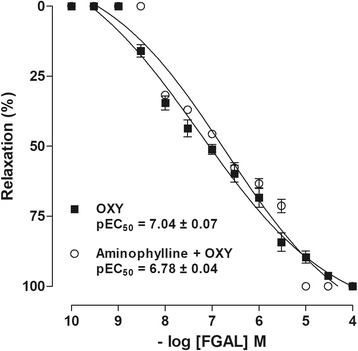
FGAL effect on tonic contractions induced by oxytocin in absence and presence of aminophylline. FGAL effect on tonic contractions induced by 10^−2^ IU/mL oxytocin in absence (■) and presence (◇) of aminophylline in rat uterus. Symbols and vertical bars represent the mean and SEM, respectively

FGAL (10^−9^–10^−4^ M, n = 5) completely relaxed the uterus pre-contracted with 10^−2^ IU/mL oxytocin (pEC_50_ = 7.0 ± 0.08), and its relaxant potency was not modified in the presence of aminophylline (pEC_50_ = 6.7 ± 0.03) when compared with the control curve (Fig. 6).

## Discussion

This study investigated the mechanism of the spasmolytic effect of galetin 3,6-dimethyl ether on rat uterus, which appears to occur by non-competitive pseudo-irreversible antagonism of oxytocin receptors and downstream pathway modulation, as positive modulation of K^+^ channels, with greatest specificity for BK_Ca_ subtype.

In preliminary pharmacological screening, it has been shown that FGAL inhibited both oxytocin- and carbachol-induced phasic contractions in the rat uterus [12]. Thus, we decided to investigate whether the flavonoid would relax the organ pre-contracted by 60 mM KCl, which induces contraction due to electromechanical mechanism and 10^−2^ IU/mL OXY, a pharmaco-mechanical coupling agent. In a concentration-dependent manner, FGAL relaxed rat uterus pre-contracted by both oxytocin and KCl, but not equipotently, being more potent in relaxing uterus pre-contracted with oxytocin.

Because FGAL was more potent in inhibiting the contractions induced with oxytocin than with carbachol [12] and because it relaxed the rat uterus pre-contracted with this agonist more potently than with KCl, a cumulative concentration-response curves to this agonist were obtained to evaluate a possible antagonism of these receptors. FGAL inhibited oxytocin-cumulative curves, and these were shifted to the right, in a non-parallel manner, with a reduction and abolition of E_max_, excluding a competitive antagonism. In addition, the non-competitive antagonism was confirmed, as the slope value (0.75 ± 0.16) was different from the unit [17].

Non-competitive antagonism occurs when the antagonist binds to the same agonist binding site (pseudo-irreversible antagonism) or in a separate site (allosteric antagonism) [24]. In addition, pseudo-irreversible antagonism occurs with a slow dissociation of the drug from the receptor, leading to a prolonged effect and abolishing the maximum effect of the agonist, differing from those observed in the allosteric antagonism, in which the maximum effect is not suppressed [25]. Thus, the profile of the cumulative concentration-response curves to oxytocin represents a pseudo-irreversible antagonism because the inhibition of the contractile response to oxytocin in the presence of FGAL has not reached a limiting value, a characteristic observed in allosteric antagonism. Additionally, it is not excluded the modulation of downstream pathway, as voltage-gated Ca^2+^ channels or K^+^ channels.

In rat uterus, extracellular and intracellular Ca^2+^ sources are important for muscular tonus development, and the Ca^2+^ entry in smooth muscle cells occurs primarily through voltage-gated Ca^2+^ channels (Ca_V_) [26]. Because the opening of these channels is a common step in the oxytocin and KCl signaling pathways to maintain the tonic phase of contraction [27] and because FGAL relaxed the rat uterus pre-contracted by both contractile agents, it was hypothesized that FGAL could be acting by blocking the Ca^2+^ influx through Ca_V_. Therefore, cumulative contractions were induced with CaCl_2_ in depolarizing medium, nominally without Ca^2+^_,_ in both the absence and the presence of different concentrations of FGAL. The flavonoid antagonized the CaCl_2_-induced contractions, as evidenced by the shift of the control curve to the right, in a non-parallel manner, with reduced E_max_, indicating that the flavonoid inhibits Ca^2+^ influx through the Ca_V_.

The K^+^ channel plays a key role in membrane potential regulation and can modulate the openness of Ca_V_, regulating the Ca^2+^ influx in smooth muscle cells [28]. Substances able to open K^+^ channels, such as cromacalin and pinacidil, comprise a diverse group of molecules with therapeutic potential by preventing the cell excitation [29]. These compounds open K^+^ channels, resulting in hyperpolarization by increasing K^+^ efflux, and consequently reduce the cytosolic concentration of Ca^2+^ ([Ca^2+^]_c_), followed by smooth muscle relaxation [30, 31]. Hence, we decided to investigate the possible positive modulation of these channels using the CsCl, a non-selective K^+^ channel blocker as pharmacological tool [18]. The relaxant potency of FGAL was reduced approximately 22-fold in the presence of CsCl, confirming the positive modulation of K^+^ channels by FGAL.

In uterine smooth muscle, the most abundant and most studied subtypes of K^+^ channels are BK_Ca_, SK_Ca_, K_V_ and K_ATP_ [32]. Accordingly, we decided to investigate the involvement of these subtypes of K^+^ channels in the tocolytic action of FGAL using selective blockers. The relaxation curve induced by FGAL was shifted to the right in the presence of 4-aminopyridine (36-fold), a selective K_V_ blocker [19], glibenclamide (50-fold), a selective K_ATP_ blocker [20], apamin (4-fold), a selective SK_Ca_ blocker [21], and 1 mM TEA^+^ (100-fold), a BK_Ca_ blocker [22]. These results indicate that these subtypes of K^+^ channels are involved in the FGAL tocolytic mechanism of action on the rat uterus. Moreover, in the presence of 1 mM TEA^+^, the relaxant potency of FGAL was more reduced than in the presence of the other blockers, indicating that FGAL primarily positively modulates BK_Ca_, which is important because it has been described that uterine smooth muscle presents greater expression of these channels compared with the other subtypes and that they play a fundamental role in regulating the basal tone of this organ [33].

Another important pathway involved in smooth muscle relaxation is the cyclic nucleotide phosphodiesterases (PDEs) pathway. Cyclic monophosphates of adenosine and guanosine (cAMP and cGMP, respectively) are able to activate protein kinase A (PKA) and protein kinase G (PKG), respectively, when occurs an elevation in the concentration of cAMP and cGMP in the cytosolic medium. These kinases can phosphorylate several intracellular targets, leading to smooth muscle relaxation, and one of these targets are K^+^ channels. PDE is responsible for cAMP and cGMP hydrolysis, resulting in their inactive products 5′-AMP and 5′-GMP, respectively, which do not activate PKA and PKG, thus stopping the cell signaling mechanism dependent on cyclic nucleotides [23]. Substances that raise the intracellular content of cAMP or cGMP show a potential relaxant effect through PDE inhibition in different tissues, including uterine smooth muscle [23].

Thus, to evaluate the involvement of this pathway, aminophylline, a non-selective PDE inhibitor [23], was used as a pharmacologic tool. The FGAL relaxant potency was not altered in both the absence and the presence of aminophylline, suggesting that the cyclic nucleotide PDEs pathway is not involved on relaxing effect induced by FGAL.

## Conclusion

In conclusion, the FGAL tocolytic action mechanism in rat uterus involves a non-competitive pseudo-irreversible antagonism of oxytocin receptors and a positive modulation of K^+^ channels, primarily the BK_Ca_ channels subtype, which indirectly modulates the Ca_V_, leading to a reduction in Ca^2+^ influx and uterine smooth muscle relaxation. Thereby, FGAL appears promising for the treatment of disorders affecting the uterine smooth muscle, such as the pain caused by colic and dysmenorrhea. However, more research is needed to better elucidate the FGAL tocolytic action mechanism in the rat uterus, such as molecular investigation of ion flow through the channels modulated by FGAL and the effect of FGAL on cytosolic calcium concentration of uterus myocytes.

## Abbreviations

BK_Ca_: Big-conductance Ca^2+^-activated K^+^ channel
FGAL: Flavonoid galetin 3,6 dimethyl ether
K_ATP_: ATP-sensitive K^+^ channel
OXY: Oxytocin
PDEs: Phosphodiesterases
SK_Ca_: Small-conductance Ca^2+^-activated K^+^ channel
